# Genetic variants of *MTHFR* gene in relation to folic acid levels and bone mineral density in Polish patients with inflammatory bowel disease

**DOI:** 10.1007/s13353-023-00792-6

**Published:** 2023-10-11

**Authors:** Alicja E. Ratajczak-Pawłowska, Szymon Hryhorowicz, Aleksandra Szymczak-Tomczak, Ewa Wysocka, Michał Michalak, Marta Kaczmarek-Ryś, Emilia Lis-Tanaś, Lena Bielawska, Andrzej Pławski, Ryszard Słomski, Agnieszka Dobrowolska, Iwona Krela-Kaźmierczak

**Affiliations:** 1 Department of Gastroenterology, Dietetics and Internal Diseases, Poznan University of Medical Sciences, Przybyszewskiego 49, 60-355 Poznan, Poland; 2 Doctoral School, Poznan University of Medical Sciences, Bukowska 70, 60-812 Poznan, Poland; 3 Institute of Human Genetics, Polish Academy of Sciences Poznan, Strzeszynska 32, 60-479 Poznan, Poland; 4 Department of Laboratory Diagnostics, Poznan University of Medical Sciences, Szamarzewskiego 82/84, 60-569 Poznan, Poland; 5 Department of Computer Science and Statistics, Poznan University of Medical Sciences, Rokietnicka 7, 60-806 Poznan, Poland

**Keywords:** Bone mineral density, Inflammatory bowel disease, Folic acid, *MTHFR*, Osteoporosis

## Abstract

Lower bone mineral density (BMD) constitutes a common issue in inflammatory bowel disease (IBD). Studies often explore the association between BMD and folic acid level. The presented study aimed to evaluate the impact of *MTHFR* gene polymorphism and folic acid levels on BMD in patients with IBDs: Crohn’s disease (CD) and ulcerative colitis (UC). The study group comprised IBD patients and a healthy control group. BMD, T-score, and Z-score of the lumbar spine (L1-L4) and femoral neck (FN) were assessed using dual-energy X-ray absorptiometry. Folic acid level was determined using direct chemiluminescence, and the *MTHFR* 677C > T (rs1801133) and 1298A > C (rs1801131) genotyping were performed by HRMA. Our study found no significant differences in the folic acid levels between the groups. Patients with CD and UC presented a lower BMD, T-score, and Z-score of the FN and L1-L4 than the CG. UC patients who were homozygotes AA in loci c.1298A>C presented lower than controls lumbar spine L1-L4 BMD and T-score values. Regarding *MTHFR* 677 polymorphism, we found that IBD patients carrying CC genotype demonstrated lower than controls femoral neck Z-score, lumbar spine L1-L4 BMD, T-score and Z-score. *MTHFR* polymorphisms were found to have no impact on folic acid concentrations. IBD patients presented a higher risk of low BMD than the healthy controls, regardless of *MTHFR* 677 and 1298 genotypes. However, *MTHFR* polymorphism may influence on bone in IBD patients. Nevertheless, it appears essential to conduct further studies.

**Supplementary Information:**

The online version contains supplementary material available at 10.1007/s13353-023-00792-6.

## Introduction

Lower bone mineral density (BMD), leading to osteopenia and osteoporosis, represents a common problem in inflammatory bowel disease (IBD), particularly among young patients (Jois et al. 2022; Kärnsund et al. 2020). It is worth bearing in mind that a reduced BMD increases the risk of fractures and is associated with a decreased quality of life (Lima et al. 2017). Therefore, it is vital to investigate new risk factors associated with osteopenia and osteoporosis in IBD, such as nutritional deficiencies and genetic aspects.

Folic acid (vitamin B9) deficiency constitutes one of the most common issues in IBD, including Crohn’s disease (CD) and ulcerative colitis (UC) (Weisshof and Chermesh 2015). Folate deficiency is also common, particularly among patients treated with sulfasalazine. Interestingly, folate deficiency was reported as more frequent among CD patients than in UC or healthy subjects (Yakut et al. 2010). Moreover, newly diagnosed pediatric IBD patients also present lower red blood cells and whole-blood folate than the control group (Heyman et al. 2009).

Folic acid plays various roles in the human body. It is essential for biological methylation and participates in nucleotide synthesis. Additionally, folate promotes the conversion of homocysteine to methionine (Gioxari et al. 2020), whereas folic acid may also inhibit colorectal carcinogenesis (Liang et al. 2021), which seems crucial for UC patients. However, folic acid supplementation in IBD patients is not routinely recommended by the European Crohn’s and Colitis Organisation (Raine et al. 2022). In terms of folic acid deficiency, it leads to anemia, hyperhomocysteinemia, and DNA damage (Ratajczak et al. 2021). According to one of the studies, hyperhomocysteinemia in CD patients was negatively correlated with folate levels (Chowers et al. 2000). Additionally, another report suggested that homocysteine and folic acid levels may affect BMD (Narváez et al. 2020).

Nutritional deficiency in IBD is multifactorial and includes decreased oral intake, malabsorption, higher nutritional requirements, and drug interaction (Lucendo and Rezende 2009). Moreover, the folic acid level also depends on *MTHFR* gene (methylenetetrahydrofolate reductase) variability. According to Colson et al., the *MTHFR* 677TT genotype was associated with a higher homocysteine concentration and a reduced folic acid concentration (Colson et al. 2017). Additionally, the TT genotype was also associated with a lower BMD among postmenopausal women (Li and Wu 2010).

Many studies discuss the relationship between BMD and folic acid level (Bailey et al. 2015; Salari et al. 2014; Kalimeri et al. 2020). However, no studies up to date have focused on the association between BMD, *MTHFR* gene polymorphism, homocysteine, and folic acid levels in inflammatory bowel disease.

Therefore, the aim of the presented study was to investigate the impact of *MTHFR* gene polymorphism and folic acid level on bone mineral density among patients suffering from CD and UC.

## Materials and methods

### Study groups

The study group included patients with IBD (Crohn’s disease and ulcerative colitis) diagnosed according to the radiological, histopathological, and endoscopic criteria. The patients were diagnosed and treated at the Department of Gastroenterology, Dietetics and Internal Diseases at Poznan University of Medical Sciences. Additionally, healthy adults were enrolled as a control group (CG). Patients under 18 or over 50 years of age, pregnant, and with diseases affecting BMD (such as diabetes, celiac disease, rheumatoid arthritis, endocrine disorders, liver disease, chronic kidney disease, chronic obstructive pulmonary disease, active neoplastic disease, other severe disorders, immunological diseases, and ongoing chronic inflammatory processes), as well as individuals treated with sulfasalazine were excluded from the study. IBD patients were treated according to the recommendations of the European Crohn’s and Colitis Organisation and the Polish Society of Gastroenterology.

### Dual-energy X-ray absorptiometry

Bone mineral density, T-score, and Z-score of the lumbar spine (L1-L4) and femoral neck (FN) were assessed using dual-energy X-ray absorptiometry with Lunar DPX-Plus (Lunar, Inc., Madison, Wisconsin, USA).

### Laboratory tests and genetic analysis

Folic acid level was determined by direct chemiluminescence (Atellica analyzers; Siemens Healthcare Diagnostics Inc., USA).

DNA was extracted from the whole-blood leukocytes using guanidine isothiocyanate and phenol–chloroform extraction. Isolates were dissolved in 1xTE buffer and stored at − 20 °C until use. The *MTHFR* 677 C > T (rs1801133) and 1298A > C (rs1801131) genotyping was performed using HRMA. The sequences of the primers used in the analysis were designed by the Pirmer3Plus program. The following primers were used: Forward 5′-CTTTGAGGCTGACCTGAAGC, Reverse 5′-AGGACGGTGCGGTGAGAGTG for 677 C > T, and Forward 5′-CTTTGGGGAGCTGAAGGACTACTAC, Reverse CACTTTGTGACCATTCCGGTTTG for 1298 > C.

### Statistical analysis

Data were presented as mean, and standard deviation (mean ± SD). The comparison between the three groups (CD vs. UC vs. CG) was performed by either the analysis of variance (ANOVA), or the Kruskal–Wallis test. The post hoc analysis was performed by the Tukey test and Dunn’s test. Categorical data were presented as numbers and percentages, and analyzed according to the chi-square test for independence. When comparing two groups (IBD vs. CG), the *t*-student test, or the Mann–Whitney test was applied. The analysis was performed using TIBCO Software Inc.'s statistical package (2017), and Statistica (data analysis software system), version 13. http://statistica.io. All tests were considered significant at *P-*value < 0.05.

## Results

The study included 90 patients with IBD (47 patients with CD and 43 with UC) and 40 healthy subjects. No significant differences between the groups were observed in age and folic acid levels. Patients with IBD presented a lower BMD, T-score, and Z-score of the femoral neck and L1-L4 lumbar spine compared to the CG. They also presented higher CRP (C-reactive protein) concentrations than healthy adults (Table 1).

**Table 1:** Characteristics of the study group

	CD (*n* = 47)	UC (*n* = 43)	CG (*n* = 40)	*P*-value
Sex, n (%)				
Women	25 (53.19%)	22 (51.16%)	22 (55.00%)	0.94
Men	22 (46.81%)	21 (48.84%)	18 (45.00%)	
Parameters; mean ± SD				
Age, years	32.89 ± 8.27	32.69 ± 9.11	36.67 ± 8.09	0.07
Femoral neck, BMD, g/cm2	1.017 ± 0.181	1.012 ± 0.188	1.121 ± 0.110	< 0.001 < 0.01^a^ < 0.01^b^ 0.99^c^
Femoral neck, T-score	− 0.26 ± 1.33	− 0.29 ± 1.41	0.52 ± 0.82	< 0.01 < 0.01^a^ < 0.01^b^ 0.99^c^
Femoral neck, Z-score	0.07 ± 1.38	0.04 ± 1.31	0.79 ± 0.84	< 0.01 < 0.01^a^ < 0.01^b^ 0.99^c^
Lumbar spine, L1-L4, BMD, g/cm2	1.140 ± 0.142	1.120 ± 0.207	1.256 ± 0.121	< 0.001 < 0.01^a^ < 0.01^b^ 0.99^c^
Lumbar spine, L1-L4, T-score	− 0.49 ± 1.22	− 0.54 ± 1.45	0.52 ± 1.01	< 0.001 < 0.01^a^ < 0.01^b^ 0.99^c^
Lumbar spine, L1-L4, Z-score	− 0.27 ± 1.16	− 0.30 ± 1.40	0.51 ± 0.98	< 0.01 < 0.01^a^ 0.02^b^ 0.99^c^
Folic acid concentration, ng//l	9.19 ± 14.13	16.08 ± 31.69	8.55 ± 3.64	0.04 0.07^a^ 0.99^b^ 0.10^c^
CRP, mg/l	25.28 ± 38.92	20.85 ± 32.28	1.20 ± 1.24	< 0.0001 < 0.0001^a^ < 0.0001^b^ 0.99^c^

*BMD*, bone mineral density; *CD*, Crohn’s disease; *CG*, control group; *CRP*, C-reactive protein; *IBD*, inflammatory bowel disease; *UC*, ulcerative colitis; a-CD vs CG; b-UC vs CG; c-CD vs UC

Regarding the alleles and genotypes frequencies, there were no significant differences between groups’ distribution of *MTHFR* 677 or *MTHFR* 1298 (Tables 2 and 3)*.*

**Table 2:** Frequencies of allele and genotypes of *MTHFR* 677

	CD (*n* = 47)	UC (*n* = 43)	CG (*n* = 40)	*P*-value
The *MTHFR* 677 genotypes frequencies [n,(%)]				
CC	26 (55.32%)	21 (48.84%)	23 (57.50%)	0.91
CT	16 (34.04%)	18 (41.86%)	14 (35.00%)	
TT	5 (10.64%)	4 (9.30%)	3 (7.50%)	
Allele frequencies in *MTHFR* 677 [n,(%)]				
C	68 (72.34%)	60 (69.77%)	60 (75.00%)	0.75
T	26 (27.66%)	26 (30.23%)	20 (25.00%)	

*CD*, Crohn’s; *CG*, control group; *IBD*, inflammatory bowel disease; *UC*, ulcerative colitis

**Table 3:** Frequencies of allele and genotypes of *MTHFR* 1298

	CD (*n* = 47)	UC (*n* = 43)	CG (*n* = 40)	*P*-value
The *MTHFR* 1298 genotypes frequencies [n,(%)]				
AA	19 (40.43%)	18 (41.86%)	20 (50.00%)	0.82
AC	20 (42.55%)	19 (44.19%)	13 (32.50%)	
CC	8 (17.02%)	6 (13.95%)	7 (17.50%)	
Allele frequencies in *MTHFR* 1298 [n,(%)]				
A	58 (61.70%)	55 (63.95%)	53 (66.25%)	0.82
C	36 (38.30%)	31 (36.05%)	27 (33.75%)	

*CD*, Crohn’s disease; *CG*, control group; *IBD*, inflammatory bowel disease; *UC*, ulcerative colitis

Moreover, no significant differences were found in BMD, T-score, and Z-score of the femoral neck and the lumbar spine and folic acid concentration depending on the *MTHFR* 677 and *MTHFR* 1298 polymorphisms (Supplementary Tables S1–S4). However, significant differences between CD, UC, and the controls were observed in BMD, T-score and Z-score of the femoral neck and of L1-L4 concerning the genotypes of *MTHFR* 677 and *MTHFR* 1298 *loci* (Tables 4–5). We noticed that significant differences were apparent in lumbar spine L1-L4 BMD and T-score. UC patients that were homozygotes AA in loci c.1298A > C presented lower than controls lumbar spine L1-L4 BMD and T-score values (1.08 ± 0.262 vs 1.284 ± 0.137, *P*-value < 0.01).

**Table 4:** Comparing bone mineral density, T-score and Z-score and folic acid level among groups taking into account genotypes of *MTHFR* 677 loci

MTHFR 677						
CC						
Parameters, mean ± SD	CD (*n* = 26)	UC (*n* = 21)	CG (*n* = 23)	IBD (*n* = 47)	*P*-value^1^	*P*-value^2^
Femoral neck, BMD, g/cm^2^	1.005 ± 0.181	0.999 ± 0.176	1.095 ± 0.086;	1.002 ± 0.177	0.06	0.02
Femoral neck, T-score	− 0.37 ± 1.33	− 0.40 ± 1.33	0.32 ± 0.65;	− 0.38 ± 1.32	0.06	0.02
Femoral neck, Z-score	− 0.14 ± 1.32	− 0.05 ± 1.32	0.64 ± 0.72	− 0.10 ± 1.31	0.02 0.02^a^ 0.19^b^ 0.99^c^	< 0.01
Lumbar spine, L1-L4, BMD, g/cm^2^	1.144 ± 0.134	1.132 ± 0.162	1.240 ± 0.113	1.138 ± 0.146	0.03 0.04^a^ 0.10^b^ 0.99^c^	< 0.01
Lumbar spine, L1-L4, T-score	− 0.48 ± 1.14	− 0.56 ± 1.42	0.38 ± 0.97	− 0.52 ± 1.26	0.03 0.04^a^ 0.09^b^ 0.99^c^	< 0.01
Lumbar spine, L1-L4, Z-score	− 0.33 ± 1.08	− 0.25 ± 1.34	0.38 ± 0.89	− 0.294 ± 1.190	0.04 0.045^a^ 0.17^b^ 0.99^c^	0.01
Folic acid concentration, ng//l	7.90 ± 6.85	11.26 ± 7.64	8.80 ± 3.70	9.40 ± 7.33	0.08	0.39
CT						
Parameters, mean ± SD	CD (*n* = 16)	UC (*n* = 18)	CG (*n* = 14)	IBD (*n* = 34)	*P*-value^1^	*P*-value^2^
Femoral neck, BMD, g/cm2	1.062 ± 0.193	1.000 ± 0.208	1.126 ± 0.083	1.029 ± 0.201	0.03 0.32^a^ 0.03^b^ 0.99^c^	0.02
Femoral neck, T-score	0.09 ± 1.42	− 0.38 ± 1.56	0.58 ± 0.58	− 0.16 ± 1.49	0.03 0.31^a^ 0.03^b^ 0.99^c^	0.02
Femoral neck, Z-score	0.57 ± 1.50	− 0.04 ± 1.35	0.81 ± 0.71	0.24 ± 1.44	0.06	0.045
Lumbar spine, L1-L4, BMD, g/cm^2^	1.149 ± 0.171	1.088 ± 0.264	1.270 ± 0.120	1.117 ± 0.224	0.06	0.02
Lumbar spine, L1-L4, T-score	− 0.39 ± 1.49	− 0.66 ± 1.60	0.67 ± 0.98	− 0.53 ± 1.53	0.06	0.02
Lumbar spine, L1-L4, Z-score	− 0.11 ± 1.42	− 0.46 ± 1.53	0.67 ± 0.97	− 0.29 ± 1.47	0.20	0.10
Folic acid concentration, ng//l	7.01 ± 4.83	23.95 ± 47.89	8.58 ± 3.89	15.97 ± 35.58	0.33	0.36
TT						
Parameters, mean ± SD	CD (*n* = 5)	UC (*n* = 4)	CG (*n* = 3)	IBD (*n* = 9)	*P*-value^1^	*P*-value^2^
Femoral neck, BMD, g/cm^2^	0.939 ± 0.123	1.138 ± 0.144	1.767 ± 1.845	1.027 ± 0.162	0.02 0.03^a^ 0.99^b^ 0.14^c^	0.06
Femoral neck, T-score	− 0.80 ± 0.91	0.68 ± 0.99	1.77 ± 1.79	− 0.14 ± 1.18	0.046 0.08^a^ 0.99^b^ 0.17^c^	0.14
Femoral neck, Z-score	− 0.46 ± 0.91	0.85 ± 1.11	1.31 ± 0.20	0.12 ± 1.16	0.10	0.20
Lumbar spine, L1-L4, BMD, g/cm^2^	1.087 ± 0.085	1.203 ± 0.118	1.314 ± 0.202	1.139 ± 0.112	0.09	0.10
Lumbar spine, L1-L4, T-score	− 0.82 ± 0.72	0.08 ± 1.05	0.80 ± 1.66	− 0.42 ± 0.95	0.18	0.13
Lumbar spine, L1-L4, Z-score	− 0.54 ± 0.54	0.18 ± 1.26	0.67 ± 1.84	− 0.22 ± 0.94	0.46	0.46
Folic acid concentration, ng//l	22.87 ± 40.62	6.00 ± 0.92	6.49 ± 1.47	15.37 ± 30.07	0.78	0.58

*BMD*, bone mineral density; *CD*, Crohn’s disease, *CG*, control group; *IBD*, inflammatory bowel disease; *UC*, ulcerative colitis; 1-CD vs UC vs CG; 2-IBD vs CG; a-CD vs CG; b-UC vs CG; c-CD vs UC

**Table 5:** Comparing bone mineral density, T-score and Z-score and folic acid level among groups taking into account genotypes of *MTHFR* 1298 loci

*MTHFR* 1298						
AA						
Parameters, mean ± SD	CD (*n* = 19)	UC (*n* = 18)	CG (*n* = 20)	IBD (*n* = 37)	*P*-value^1^	*P*-value^2^
Femoral neck, BMD, g/cm^2^	1.056 ± 0.193	1.021 ± 0.215	1.137 ± 0.128	1.039 ± 0.202	0.09	0.03
Femoral neck, T-score	0.07 ± 1.41	− 0.24 ± 1.65	0.65 ± 0.96	− 0.08 ± 1.52	0.12	0.04
Femoral neck, Z-score	0.48 ± 1.46	0.03 ± 1.53	0.88 ± 1.00	0.26 ± 1.49	0.15	0.06
Lumbar spine, L1-L4, BMD, g/cm^2^	1.164 ± 0.162	1.080 ± 0.262	1.284 ± 0.137	1.123 ± 0.217	< 0.01 0.08^a^ < 0.01^b^ 0.99^c^	< 0.01
Lumbar spine, L1-L4, T-score	− 0.23 ± 1.33	− 0.75 ± 1.63	0.74 ± 1.183	− 0.48 ± 1.48	< 0.01 0.08^a^ < 0.01^b^ 0.99^c^	< 0.01
Lumbar spine, L1-L4, Z-score	0.01 ± 1.22	− 0.54 ± 1.52	0.66 ± 1.25	− 0.26 ± 1.38	0.05 0.53^a^ 0.046^b^ 0.85^c^	0.03
Folic acid concentration, ng//l	11.21 ± 20.92	24.78 ± 47.65	8.48 ± 3.24	17.81 ± 36.58	0.13	0.22
AC						
Parameters, mean ± SD	CD (*n* = 20)	UC (*n* = 19)	CG (*n* = 13)	IBD (*n* = 39)	*P*-value^1^	*P*-value^2^
Femoral neck, BMD, g/cm^2^	0.994 ± 0.183	1.003 ± 0.171	1.089 ± 0.092	0.998 ± 0.175	0.08	0.03
Femoral neck, T-score	− 0.45 ± 1.34	− 0.34 ± 1.26	0.30 ± 0.65	− 0.40 ± 1.28	0.08	0.03
Femoral neck, Z-score	− 0.11 ± 1.32	0.04 ± 1.15	0.53 ± 0.56	− 0.04 ± 1.22	0.06	0.02
Lumbar spine, L1-L4, BMD, g/cm^2^	1.124 ± 0.134	1.158 ± 0.166	1.238 ± 0.110	1.141 ± 0.149	0.09	0.05
Lumbar spine, L1-L4, T-score	− 0.64 ± 1.20	− 0.31 ± 1.40	0.36 ± 0.83	− 0.48 ± 1.29	0.09	0.049
Lumbar spine, L1-L4, Z-score	− 0.36 ± 1.13	− 0.03 ± 1.42	0.41 ± 0.70	− 0.20 ± 1.28	0.15	0.10
Folic acid concentration, ng//l	8.63 ± 7.32	8.88 ± 5.19	9.25 ± 4.71	8.76 ± 6.29	0.65	0.49
CC						
Parameters, mean ± SD	CD (*n* = 8)	UC (*n* = 6)	CG (*n* = 7)	IBD (*n* = 14)	*P*-value^1^	*P*-value^2^
Femoral neck, BMD, g/cm^2^	0.985 ± 0.149	1.016 ± 0.185	1.133 ± 0.082	0.998 ± 0.159	0.19	0.08
Femoral neck, T-score	− 0.54 ± 1.09	− 0.28 ± 1.33	0.56 ± 0.67	− 0.43 ± 1.16	0.25	0.10
Femoral neck, Z-score	− 0.50 ± 1.17	0.05 ± 1.36	1.00 ± 0.76	− 0.26 ± 1.24	0.07	0.03
Lumbar spine, L1-L4, BMD, g/cm^2^	1.121 ± 0.122	1.119 ± 0.130	1.212 ± 0.076	1.121 ± 0.121	0.20	0.08
Lumbar spine, L1-L4, T-score	− 0.70 ± 1.05	− 0.67 ± 1.13	0.16 ± 0.69	− 0.69 ± 1.04	0.17	0.07
Lumbar spine, L1-L4, Z-score	− 0.74 ± 0.99	− 0.40 ± 0.88	0.24 ± 0.36	− 0.59 ± 0.93	0.13	0.07
Folic acid concentration, ng//l	5.75 ± 3.87	12.80 ± 11.54	7.44 ± 2.43	8.77 ± 8.51	0.31	0.43

*BMD*, bone mineral density; *CD*, Crohn’s disease, *CG*, control group; *IBD*, inflammatory bowel disease; *UC*, ulcerative colitis; 1-CD vs UC vs CG; 2-IBD vs CG; a-CD vs CG; b-UC vs CG; c-CD vs UC

Regarding *MTHFR* 677 polymorphism, we found that IBD patients carrying the CC genotype demonstrated lower than controls: femoral neck Z-score, lumbar spine L1-L4 BMD, T-score and Z-score (*P*-value ≤ 0.01). The lowest parameters were presented in CD patients, who demonstrated femoral neck Z-score − 0.14 ± 1.32 (vs 0.64 ± 0.72 in controls, *P*-value = 0.02); lumbar spine L1-L4 BMD 1.14 ± 0.13 (vs 1.24 ± 0.11, *P*-value = 0.04); lumbar spine L1-L4 T-score − 0.48 ± 1.14 (vs 0.38 ± 0.97, *P*-value = 0.04); lumbar spine L1-L4 Z-score − 0.33 ± 1.08 (vs 0.38 ± 0.89, *P*-value = 0.05).

We noticed that in the case of IBD heterozygotes CT, the lowest values of parameters were presented by UC patients; however, it was below the accepted level of significance.

There were no statistically relevant differences in folic acid concentration between groups with identical genotypes (Tables 4 and 5).

## Discussion

In our research, patients with CD and UC presented a lower BMD, T-score, and Z-score of the femoral neck and L1-L4 lumbar spine compared to the healthy subjects, which was also demonstrated in our previous research (Ratajczak et al. 2022; Krela-Kaźmierczak et al. 2018). Other studies also indicate that a lower BMD constitutes a common issue among IBD patients. Lee et al. reported that about 33% of the newly diagnosed Asian patients with IBD showed a low BMD (Lee et al. 2021). A low BMD was also an issue among children with IBD, and the factors associated with osteoporosis comprised a low BMI (body mass index), ileocolonic disease location, low hemoglobin and calcium levels, and infliximab therapy (Isa et al. 2023).

No significant differences were observed in folic acid concentrations between assessed groups, which is contrary to the meta-analysis performed by Pan et al., who demonstrated that IBD patients presented a lower folic acid concentration than the healthy controls (Pan et al. 2017). Bearing in mind the findings of Yakut et al., who reported that folic acid deficiency is more common among CD than UC patients (Yakut et al. 2010), it is reasonable to assume that part of IBD patients in our group supplemented folic acid. Folic acid concentration was non-significantly higher in the UC group than in CD or healthy subjects. We suppose that patients suffering from UC supplemented folic acid more often than CD patients or healthy adults, because folic acid supplementation may decrease the risk of colorectal cancer in this group of patients (Biasco and Marco 2005).

No significant differences were noted in the *MTHFR* 677 and 1298 genotypes frequencies between the study and control groups. Nonetheless, our study group was not numerous.

In other studies, involving *MTHFR* genotypes frequencies, results are inconsistent. For instance, the study by Stocco et al. involving young patients with IBD showed no significant differences compared to healthy subjects (Stocco et al. 2006). Conversely, in the Chinese population, heterozygous AC and homozygous CC genotypes of *MTHFR* 1298 occurred significantly more frequently among UC patients as compared to healthy adults. However, no significant differences were observed in the frequencies of *MTHFR* 677 allele (T) and genotypes (CT + TT). Additionally, heterozygotes of *MTHFR* (677CT + 1298AC) were associated with pancolitis and extra-intestinal complications in UC (Jiang et al. 2012). Data concerning the impact of *MTHFR* polymorphisms on the course of IBD also remain vague. Varzari et al. reported no strong association between *MTHFR* 677 or *MTHFR* 1298 variants and the risk of UC. However, they suggested that those variants may affect UC severity (Varzari et al. 2015). In the Moroccan cohort study, the 677CT polymorphism in *MTHFR* also did not modulate the risk of IBD (Senhaji et al. 2013).

Our study found no differences in the folic acid concentration between groups. However, Pan et al. reported that the serum folate is lower in IBD and UC, although not in CD groups, than in healthy adults (Pan et al. 2017). Moreover, folic acid level may also depend on disease activity. According to one of the studies, serum folate levels correlated negatively with CRP levels (Moein et al. 2020). It is worth bearing in mind that folate deficiency affects 3% of patients with UC and 5% of CD patients (Park et al. 2021), and according to Lupu et al., about 4% of IBD patients present folic acid deficiency (Lupu et al. 2015). In contrast, Santucci et al. reported no folate deficiency in patients diagnosed with IBD in a follow-up period of 6 months to 5 years (Santucci et al. 2014). However, it should be noted that our study did not assess folic acid supplementation, which in turn may affect folic acid concentration.

There were no differences in BMD, T-score, and Z-score depending on the *MTHFR* 677 and *MTHFR* 1298 genotypes in the study groups. However, our study demonstrated that BMD, T-score, and Z-score in IBD patients were lower than in the controls, which was not associated with *MTHFR* 677 and *MTHFR* 1298 genotypes. Nevertheless, studies on the impact of *MTHFR* polymorphism on BMD are inconclusive. According to the meta-analysis, the *MTHFR* c.677C > T variant was linked to a decreased lumbar spine and femoral neck BMD in Caucasian men and postmenopausal women. This polymorphism was also associated with a reduced total body BMD in women (Li et al. 2016). In fact, postmenopausal Thai women with the *MTHFR* 677 CT genotype presented a higher risk of osteopenia than the control group genotype (OR = 5.66; *P*-value < 0.001) (Tongboonchoo et al. 2013). In contrast, Pandey et al. did not find differences in genotypes’ frequencies for *MTHFR* 677CT and 1298AC between the subjects with a normal and decreased BMD (Pandey et al. 2013). Shin et al. also did not observe differences in BMD of the femoral neck and lumbar spine depending on the *MTHFR* 677CT genotype in men or women (Shin et al. 2013). However, it is vital to notice that *MTHFR* 1298 A > C polymorphism may account for the susceptibility to IBD, and particularly ulcerative colitis (Yang et al. 2021). In fact, there are many risk factors of osteoporosis such as chronic inflammation, malnutrition, and insufficient intake (Krela-Kaźmierczak et al. 2018), which also may affect BMD. Maybe our group was not homogenous enough in active disease or nutritional status, disturbing the results of *MTHFR* polymorphism’s impact on bone mineral density.

Our study found no significant differences regarding folic acid concentration depending on *MTHFR* 677 and *MTHFR* 1298 genotypes. Yet, Mazokopakis et al. showed that the *MTHFR* 677TT genotype was associated with a significantly lower folate level than subjects with the *MTHFR* 677 CT or CC genotype (Mazokopakis et al. 2023). Thus, supplementation or a high intake of folate may indeed affect folic acid concentration.

Although the role of *MTHFR* polymorphisms has been reported widely in the literature, particularly concerning their effect on folic acid levels in the context of postmenopausal osteoporosis, the frequency of *MTHFR* variants in Polish patients with Crohn’s disease and ulcerative colitis has not been shown to date. As inflammatory bowel diseases remain incurable, and low BMD (osteopenia and osteoporosis) that causes fractures are frequent severe complications in the IBD course, such research is necessary and relevant, particularly in the population-specific backdrop. However, our study bears certain limitations. Firstly, the group is not numerous. Secondly, the study did not address homocysteine levels, which may also be modulated by *MTHFR* variants and may affect BMD. Furthermore, the subjects in our study were not asked to provide information concerning the supplementation of folic acid and folate intake, which may have affected the resulting folic acid levels.

In conclusion, our study demonstrated no impact of *MTHFR* polymorphism on IBD’s BMD and folic acid concentration. However, IBD patients presented a higher risk of low BMD than the healthy controls, regardless of *MTHFR* 677 and 1298 genotypes. Therefore, further studies are essential, which would include more patients and assess *MTHFR* gene polymorphism, BMD, the intake and supplementation of vitamins B6 and B12, as well as their concentrations.

### Supplementary Information

Below is the link to the electronic supplementary material.

Supplementary file1 (DOCX 30 KB)

## Data Availability

The data underlying this article will be shared on reasonable request to the corresponding author.
