# Supplementary material for: Genetic variants of MTHFR gene in relation to folic acid levels and bone mineral density in Polish patients with inflammatory bowel disease
Source: J Appl Genet. 2023 Oct 11;65(1):73–81. doi: 10.1007/s13353-023-00792-6 (PMC10789830; doi:10.1007/s13353-023-00792-6)
Supplement: Supplementary file 1 — Supplementary file1 (DOCX 30 KB) [file 13353_2023_792_MOESM1_ESM.docx]

Supplementary Table 1. Comparing bone mineral density, T-score and Z-score and folic acid level depending on genotypes of MTHFR 677 and MTHFR 1298 loci in patients with Crohn's disease.

| *MTHFR* 677 | | | | | | | | | |
| --- | --- | --- | --- | --- | --- | --- | --- | --- | --- |
| Parameters, mean±SD | CC (n=26) | CT (n=16) | TT (n=5) | CT+TT (n=21) | CC+CT (n=42) | *P*-value^1^ | *P*-value^2^ | | *P*-value^3^ |
| Femoral neck, BMD, g/cm2 | 1.005±0.181 | 1.062±0.193 | 0.939±0.123 | 1.032±0.184 | 1.027±1.186 | 0.37 | 0.61 | | 0.38 |
| Femoral neck, T-score | -0.37±1.33 | 0.09±1.42 | -0.80±0.91 | -0.12±1.35 | -0.19±1.37; | 0.36 | 0.53 | | 0.36 |
| Femoral neck, Z-score | -0.14±1.32 | 0.57±1.50 | -0.46±0.91 | 0.33±1.43 | 0.13±1.42 | 0.18 | 0.25 | | 0.45 |
| Lumbar spine, L1-L4, BMD, g/cm2 | 1.144±0.134 | 1.149±0.171 | 1.087±0.085 | 1.134±0.156 | 1.146±0.147 | 0.69 | 0.82 | | 0.32 |
| Lumbar spine, L1-L4, T-score | -0.48±1.14 | -0.39±1.49 | -0.82±0.72 | -0.49±1.34 | -0.46±1.27 | 0.79 | 0.98 | | 0.43 |
| Lumbar spine, L1-L4, Z-score | -0.33±1.08 | -0.11±1.42 | -0.54±0.54 | -0.21±1.27 | -0.24±1.21 | 0.73 | 0.73 | | 0.65 |
| Folic acid concentration, ng//l | 7.90±6.85 | 7.01±4.83 | 22.87±40.62 | 10.78±19.89 | 7.56±6.11 | 0.07 | 0.49 | | 0.55 |
| *MTHFR* 1298 | | | | | | | | | |
| Parameters, mean±SD | AA (n=19) | AC (n=20) | CC (n=8) | AC+CC (n=28) | AA+AC (n=39) | *P*-value^4^ | *P*-value^5^ | *P*-value^6^ | |
| Femoral neck, BMD, g/cm2 | 1.056±0.193 | 0.994±0.183 | 0.985±0.149 | 0.991±0.171 | 1.024±0.188 | 0.50 | 0.24 | 0.63 | |
| Femoral neck, T-score | 0.07±1.41 | -0.45±1.34 | -0.54±1.09 | -0.48±1.25 | -0.20±1.38 | 0.39 | 0.17 | 0.57 | |
| Femoral neck, Z-score | 0.48±1.46 | -0.11±1.32 | -0.50±1.17 | -0.22±1.27 | 0.18±1.40 | 0.18 | 0.08 | 0.25 | |
| Lumbar spine, L1-L4, BMD, g/cm2 | 1.164±0.162 | 1.124±0.134 | 1.121±0.122 | 1.123±0.128 | 1.144±0.147 | 0.64 | 0.34 | 0.69 | |
| Lumbar spine, L1-L4, T-score | -0.23±1.33 | -0.64±1.20 | -0.70±1.05 | -0.657±1.139 | -0.44±1.26 | 0.51 | 0.25 | 0.51 | |
| Lumbar spine, L1-L4, Z-score | 0.01±1.22 | -0.36±1.13 | -0.74±0.99 | -0.48±1.09 | -0.18±1.17 | 0.29 | 0.16 | 0.13 | |
| Folic acid concentration, ng//l | 11.21±20.92 | 8.63±7.32 | 5.75±3.87 | 7.81±6.58 | 9.89±15.36 | 0.65 | 0.42 | 0.44 | |

BMD- bone mineral density; 1-CC vs CT vs TT; 2-CT+TT vs CC; 3- CC+CT vs TT; 4- AA vs AC vs CC; 5- CC+AC vs AA; 6- AA+AC vs CC

Supplementary Table 2. Comparing bone mineral density, T-score and Z-score and folic acid level depending on genotypes of MTHFR 677 and MTHFR 1298 loci in patients with ulcerative colitis

| *MTHFR* 677 | | | | | | | | |
| --- | --- | --- | --- | --- | --- | --- | --- | --- |
| Parameters, mean±SD | CC (n=21) | CT (n=18) | TT (n=4) | CT+TT (n=22) | CC+CT (n=39) | *P*-value^1^ | *P*-value^2^ | *P*-value^3^ |
| Femoral neck, BMD, g/cm2 | 0.999±0.176 | 1.000±0.208 | 1.138±0.144 | 1.024±0.203 | 0.999±0.189 | 0.38 | 0.66 | 0.20 |
| Femoral neck, T-score | -0.40±1.33 | -0.38±1.56 | 0.68±0.99 | -0.19±1.51 | -0.39±1.42 | 0.36 | 0.62 | 0.16 |
| Femoral neck, Z-score | -0.05±1.32 | -0.04±1.35 | 0.85±1.11 | 0.12±1.34 | -0.05±1.32 | 0.44 | 0.68 | 0.19 |
| Lumbar spine, L1-L4, BMD, g/cm2 | 1.132±0.162 | 1.088±0.264 | 1.203±0.118 | 1.11±0.25 | 1.111±0.213 | 0.58 | 0.72 | 0.46 |
| Lumbar spine, L1-L4, T-score | -0.56±1.42 | -0.66±1.60 | 0.08±1.05 | -0.53±1.52 | -0.61±1.48 | 0.67 | 0.94 | 0.43 |
| Lumbar spine, L1-L4, Z-score | -0.25±1.34 | -0.46±1.53 | 0.18±1.26 | -0.34±1.48 | -0.35±1.42 | 0.71 | 0.84 | 0.44 |
| Folic acid concentration, ng//l | 11.26±7.64 | 23.95±47.89 | 6.00±0.92 | 20.68±43.67 | 17.12±33.13 | 0.38 | 0.34 | 0.37 |
| *MTHFR* 1298 | | | | | | | | |
| Parameters, mean±SD | AA (n=18) | AC (n=19) | CC (n=6) | AC+CC (n=25) | AA+AC (n=37) | *P*-value^4^ | *P*-value^5^ | *P*-value^6^ |
| Femoral neck, BMD, g/cm2 | 1.021±0.215 | 1.003±0.171 | 1.016±0.185) | 1.006±0.171 | 1.012±0.191 | 0.96 | 0.80 | 0.93 |
| Femoral neck, T-score | -0.24±1.65 | -0.34±1.26 | -0.28±1.33 | -0.33±1.25 | -0.29±1.44 | 0.98 | 0.85 | 0.97 |
| Femoral neck, Z-score | 0.03±1.53 | 0.04±1.15 | 0.05±1.36 | 0.04±1.17 | 0.04±1.32 | 0.99 | 0.98 | 0.97 |
| Lumbar spine, L1-L4, BMD, g/cm2 | 1.080±0.262 | 1.158±0.166 | 1.119±0.130 | 1.149±0.156 | 1.120±0.218 | 0.53 | 0.29 | 0.67 |
| Lumbar spine, L1-L4, T-score | -0.75±1.63 | -0.31±1.40 | -0.67±1.13 | -0.40±1.33 | -0.52±1.51 | 0.65 | 0.44 | 0.61 |
| Lumbar spine, L1-L4, Z-score | -0.54±1.52 | -0.03±1.42 | -0.40±0.88 | -0.12±1.31 | -0.28±1.47 | 0.54 | 0.33 | 0.82 |
| Folic acid concentration, ng//l | 24.78±47.65 | 8.88±5.19 | 12.80±11.54 | 9.82±7.13 | 16.61±33.92 | 0.31 | 0.13 | 0.90 |

BMD- bone mineral density; 1-CC vs CT vs TT; 2-CT+TT vs CC; 3- CC+CT vs TT; 4- AA vs AC vs CC; 5- CC+AC vs AA; 6- AA+AC vs CC

Supplementary Table 3. Comparing bone mineral density, T-score and Z-score and folic acid level depending on genotypes of MTHFR 677 and MTHFR 1298 loci in the control group

| *MTHFR* 677 | | | | | | | | | |
| --- | --- | --- | --- | --- | --- | --- | --- | --- | --- |
| Parameters, mean±SD | | CC (n=23) | CT (n=14) | TT (n=3) | CT+TT (n=17) | CC+CT (n=37) | *P*-value^1^ | *P*-value^2^ | *P*-value^3^ |
| Femoral neck, BMD, g/cm2 | | 1.095±0.086 | 1.126±0.083 | 1.767±1.845 | 1.156±0.131 | 1.107±0.085 | 0.11 | 0.12 | 0.07 |
| Femoral neck, T-score | | 0.32±0.65 | 0.58±0.58 | 1.77±1.79 | 0.79±0.96 | 0.42±0.63 | 0.13 | 0.09 | 0.11 |
| Femoral neck, Z-score | | 0.64±0.72 | 0.81±0.71 | 1.31±0.20 | 0.98±0.97 | 0.71±0.71 | 0.38 | 0.24 | 0.29 |
| Lumbar spine, L1-L4, BMD, g/cm2 | | 1.240±0.113 | 1.270±0.120 | 1.314±0.202 | 1.278±0.131 | 1.252±0.115 | 0.59 | 0.31 | 0.72 |
| Lumbar spine, L1-L4, T-score | | 0.38±0.97 | 0.67±0.98 | 0.80±1.66 | 0.69±1.06 | 0.49±0.97 | 0.61 | 0.36 | 0.98 |
| Lumbar spine, L1-L4, Z-score | | 0.38±0.89 | 0.67±0.97 | 0.67±1.84 | 0.67±1.09 | 0.49±0.92 | 0.61 | 0.43 | 0.78 |
| Folic acid concentration, ng//l | | 8.80±3.70 | 8.58±3.89 | 6.49±1.47 | 19.11±11.97 | 8.72±3.72 | 0.58 | 0.55 | 0.33 |
| *MTHFR* 1298 | | | | | | | | | |
| Parameters, mean±SD | AA (n=20) | | AC (n=13) | CC (n=7) | AC+CC (n=20) | AA+AC (n=33) | *P*-value^4^ | *P*-value^5^ | *P*-value^6^ |
| Femoral neck, BMD, g/cm2 | 1.137±0.128 | | 1.089±0.092 | 1.133±0.082 | 1.105±0.089 | 1.118±0.116 | 0.36 | 0.47 | 0.47 |
| Femoral neck, T-score | 0.65±0.96 | | 0.30±0.65 | 0.56±0.67 | 0.39±0.65 | 0.50±0.86 | 0.42 | 0.47 | 0.53 |
| Femoral neck, Z-score | 0.88±1.00 | | 0.53±0.56 | 1.00±0.76 | 0.70±0.66 | 0.74±0.86 | 0.31 | 0.54 | 0.35 |
| Lumbar spine, L1-L4, BMD, g/cm2 | 1.284±0.137 | | 1.238±0.110 | 1.212±0.076 | 1.229±0.098 | 1.266±0.127 | 0.43 | 0.20 | 0.48 |
| Lumbar spine, L1-L4, T-score | 0.74±1.183 | | 0.36±0.83 | 0.16±0.69 | 0.29±0.77 | 0.59±1.06 | 0.68 | 0.39 | 0.59 |
| Lumbar spine, L1-L4, Z-score | 0.66±1.25 | | 0.41±0.70 | 0.24±0.36 | 0.35±0.60 | 0.56±1.06 | 0.94 | 0.77 | 0.79 |
| Folic acid concentration, ng//l | 8.48±3.24 | | 9.25±4.71 | 7.44±2.43 | 8.62±4.08 | 8.79±3.83 | 0.77 | 0.99 | 0.55 |

BMD- bone mineral density; 1-CC vs CT vs TT; 2-CT+TT vs CC; 3- CC+CT vs TT; 4- AA vs AC vs CC; 5- CC+AC vs AA; 6- AA+AC vs CC

Supplementary Table 4. Comparing bone mineral density, T-score and Z-score and folic acid level depending on genotypes of MTHFR 677 and MTHFR 1298 loci in patients with inflammatory bowel disease

| *MTHFR* 677 | | | | | | | | |
| --- | --- | --- | --- | --- | --- | --- | --- | --- |
| Parameters, mean±SD | CC (n=47) | CT (n=34) | TT (n=9) | CT+TT (n=43) | CC+CT (n=81) | *P*-value^1^ | *P*-value^2^ | *P*-value^3^ |
| Femoral neck, BMD, g/cm2 | 1.002±0.177 | 1.029±0.201 | 1.027±0.162 | 1.028±0.192 | 1.014±0.187 | 0.80 | 0.50 | 0.79 |
| Femoral neck, T-score | -0.38±1.32 | -0.16±1.49 | -0.14±1.18 | -0.15±1.42 | -0.29±1.39 | 0.73 | 0.43 | 0.74 |
| Femoral neck, Z-score | -0.10±1.31 | 0.24±1.44 | 0.12±1.16 | 0.22±1.37 | 0.04±1.36 | 0.52 | 0.26 | 0.77 |
| Lumbar spine, L1-L4, BMD, g/cm2 | 1.138±0.146 | 1.117±0.224 | 1.139±0.112 | 1.121±0.0.20 | 1.129±0.181 | 0.85 | 0.64 | 0.85 |
| Lumbar spine, L1-L4, T-score | -0.52±1.26 | -0.53±1.53 | -0.42±0.95 | -0.51±1.42 | -0.52±1.37 | 0.98 | 0.98 | 0.99 |
| Lumbar spine, L1-L4, Z-score | -0.294±1.190 | -0.29±1.47 | -0.22±0.94 | -0.28±1.37 | -0.30±1.31 | 0.98 | 0.95 | 0.88 |
| Folic acid concentration, ng//l | 9.40±7.33 | 15.97±35.58 | 15.37±30.07 | 15.85±34.16 | 12.16±23.74 | 0.46 | 0.21 | 0.41 |
| *MTHFR* 1298 | | | | | | | | |
| Parameters, mean±SD | AA (n=37) | AC (n=39) | CC (n=14) | AC+CC (n=53) | AA+AC (n=76) | *P*-value^4^ | *P*-value^5^ | *P*-value^6^ |
| Femoral neck, BMD, g/cm2 | 1.039±0.202 | 0.998±0.175 | 0.998±0.159 | 0.998±0.169 | 1.018±0.188 | 0.59 | 0.30 | 0.89 |
| Femoral neck, T-score | -0.08±1.52 | -0.40±1.28 | -0.43±1.16 | -0.41±1.24 | -0.24±1.40 | 0.55 | 0.27 | 0.85 |
| Femoral neck, Z-score | 0.26±1.49 | -0.04±1.22 | -0.26±1.24 | -0.10±1.22 | 0.11±1.36 | 0.39 | 0.21 | 0.34 |
| Lumbar spine, L1-L4, BMD, g/cm2 | 1.123±0.217 | 1.141±0.149 | 1.121±0.121 | 1.135±0.141 | 1.132±0.184 | 0.89 | 0.75 | 0.59 |
| Lumbar spine, L1-L4, T-score | -0.48±1.48 | -0.48±1.29 | -0.69±1.04 | -0.53±1.23 | -0.48±1.38 | 0.87 | 0.86 | 0.45 |
| Lumbar spine, L1-L4, Z-score | -0.26±1.38 | -0.20±1.28 | -0.59±0.93 | -0.30±1.20 | -0.23±1.32 | 0.61 | 0.87 | 0.21 |
| Folic acid concentration, ng//l | 17.81±36.58 | 8.76±6.29 | 8.77±8.51 | 8.76±6.86 | 13.16±26.14 | 0.22 | 0.08 | 0.51 |

BMD- bone mineral density; 1-CC vs CT vs TT; 2-CT+TT vs CC; 3- CC+CT vs TT; 4- AA vs AC vs CC; 5- CC+AC vs AA; 6- AA+AC vs CC
